# Neonatal Mastitis: A Clinico-Microbiological Study

**Published:** 2014-01-01

**Authors:** Talat Masoodi, Gowhar Nazir Mufti, Javeed Iqbal Bhat, Rubina Lone, Syed Arshi, Syed Khurshid Ahmad

**Affiliations:** 1 Department of Microbiology, Sher-i-Kashmir Institute of Medical Sciences, Medical College, Bemina, Kashmir, India; 2 Department of Pediatric Surgery, Sher-i-Kashmir Institute of Medical Sciences, Soura, Srinagar, India; 3 Department of Neonatology, Sher-i-Kashmir Institute of Medical Sciences, Soura, Srinagar, India

**Keywords:** Mastitis, Neonate, Study

## Abstract

Objective:Neonatal breast hypertrophy is a common phenomenon in term infants, superadded infection can lead to mastitis and that can progress to breast abscess with short and long term detrimental effects. Our effort is to study the prevalence, risk factors, the current microbial profile and sensitivity pattern in these infections in order to suggest an optimal treatment plan for these patients.

Design: Case series.

Setting: Hospital based study conducted in Kashmir on the native population.

Duration: 2011 to 2013.

Materials and Methods: 32 neonates with features of mastitis or abscess were included in the study. Demographic and clinical data, laboratory work-up were recorded for all these patients in a patient form. Gram stain of the purulent nipple discharge or pus obtained on drainage was done and the specimens were culture plated. Antibiotic sensitivity was determined by disk diffusion and categorized by current Clinical and Laboratory Standards Institute (CLSI) guidelines.

Results:Most babies were full term, the age range was 6-48 days. Peak incidence for mastitis was in the 2nd week and for abscess in the 4th week. The ratio of male: female was 1:2 in the entire group, there was greater preponderance of female involvement with increasing age. Massage for expression of secretions a common practice in the study population had been done in 15 patients, especially in male babies. The babies were generally well and associated skin pustulosis was common. Laboratory workup showed polymorphonuclear leucocytosis and CRP positivity. Gram staining showed gram positive cocci in 13 patients and gram negative rods in 1 patient. Culture revealed Staphylococcus aureus in 18, E.col in 2, klebsiella in 1 patient and was sterile in 2 patients. Most strains of Staphylococcus aureus were resistant to macrolides and penicillins. Fifteen were methicillin sensitive and 3 were resistant but were sensitive to amikacin, ofloxacin and vancomycin. Gram negative rods were sensitive to, aminoglycosides, chloramphenicol, quinolones, piperacillin-tazobactum and cefoperazone-sulbactum, but were resistant to cephalosporins including third generation cephalosporins. Treatment with oral antibiotic was not successful. Patients responded well to open drainage via a stab incision away from the breast mound; 4 patients were managed by repeated needle aspirations. IV antibiotics were prescribed in all patients for 2-5 days, followed by oral continuation therapy of 7-14 days.

Conclusion: From our study, we can conclude that parental counseling to avoid massage, and early treatment for pustulosis is important to prevent mastitis. Intravenous antibiotics should be used for this condition guided by gram stain or culture sensitivity once available. Empirically a drug with good anti-staph cover may be instituted till appropriate reports are available. Incision drainage gives uniformly good results, though; multiple sittings of needle drainage may obviate the need for incision drainage. Therapy can be shifted to oral drugs once clinical improvement is seen.

## INTRODUCTION

Neonatal breast enlargement is a common response which occurs under the influence of falling levels of maternal estrogen at the end of pregnancy, triggering the release of prolactin from the newborn’s pituitary. (1) Neonatal breast enlargement is common (seen in approximately 70% of neonates) usually occurs in term infants in the first week of life and is independent of the sex of the baby. (2) Some of these enlarged breasts may discharge liquid (witch’s milk). The condition is self-limiting and needs observation and reassurance of the parents. (3) Only occasionally the hypertrophied breast bud develops an infective pathology the neonatal mastitis, with nearly 50% of these progressing to frank breast abscess. (3-6)This is more common in girls, with a male: female ratio of approximately 1:2-3.5. (3-6)This is related to the fact that physiologic breast hypertrophy lasts longer in female infants. (2) The risk factors for progression to infective mastitis or abscess are not well studied.

Neonatal mastitis is usually a localized pathology, occurring in otherwise well and usually full term infants and systemic features are rare, though it may be associated with suppurative lesions elsewhere in the body. (3,4,6) Staphylococcus aureus is the causative agent in most cases , cases due to Group B Streptococci ,gram-negative enteric bacteria and anaerobes have also been reported. (3-6)

The practices followed regarding the terminology, clinical suspicion, extent of evaluation and treatment options of this poorly understood condition vary widely. (6) Our endeavour is to study the prevalence, risk factors, the current microbial profile and sensitivity pattern in these infections. Also, to suggest an optimal treatment plan for these patients. This is especially important considering the considerable parental worry and the possible effects on later breast development in female babies affected with this condition.(2, 7)

## MATERIALS AND METHODS

The study was conducted from 2011 to 2013. 32 patients with features of mastitis such as increasing tenderness, erythematous swelling, purulent discharge, fluctuation or rupture of abscess were included in the study. Demographic and clinical data noting the gestational age, sex, age at presentation, duration of symptoms, local findings, bilaterality, any associated systemic findings, skin rash were recorded for all these patients in a patient form. Complete and differential blood counts, C - reactive protein (CRP), were done in all patients. Gram stain of the purulent nipple discharge or pus obtained on drainage was done and the specimens were culture plated. Antibiotic sensitivity was determined by disk diffusion and categorized by current Clinical and Laboratory Standards Institute (CLSI) guidelines. (8)

## RESULTS

Thirty of the study patients were full-term babies and 2 were born late preterm. The detailed age and sex distribution pattern of the patients is depicted in the table. (Table 1). The age range was 6-48 days. Peak incidence for mastitis was in the 2nd week and for abscess in the 4th week. The ratio of male: female was 1:2 in the entire group and 1:2.5 in the abscess group. There was greater preponderance of female involvement with increasing age. The usual duration of symptoms was 2-3 days with a range of 1-7 days; the duration was more in patients with breast abscess.

**Figure:**
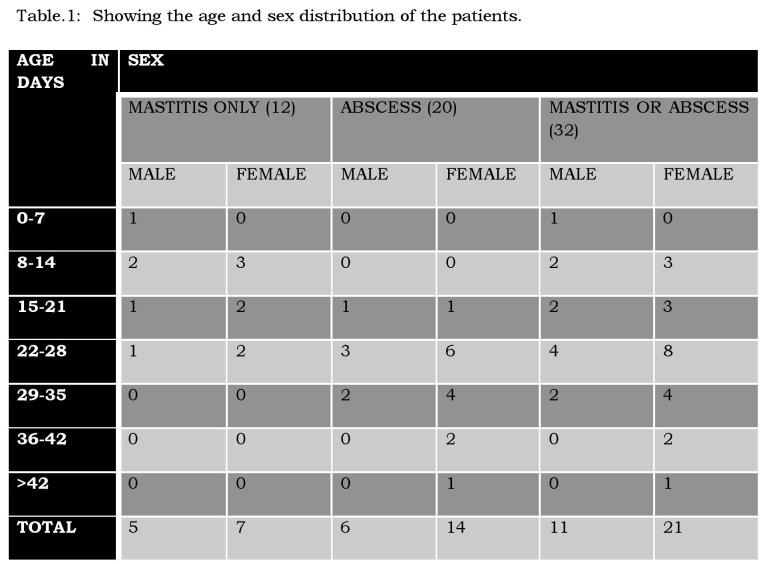
Table 1

All babies were breast fed. Massage for expression of secretions a common practice in the study population had been done in 15 patients, especially in male babies. Of the thirty two patients included in the study, 20 (63%) had frank breast abscess. The babies were generally well and systemic features were noted in only 6 patients, fever in 5 and refusal of feeds in 3. Associated skin rash, pustulosis especially in the neck, axillae, and groin was noted in 12 patients, 8 of these had breast abscess. There was bilateral involvement in 5 patients, 3 with bilateral breast abscess, 4 of these had skin pustulosis.

Polymorphonuclear leucocytosis (WBC counts more than 17000/cmm and or polymorphs >70%) was observed in 6 neonates with mastitis and in 12 babies with breast abscess. C-reactive protein (CRP) was positive in 20 patients. 

Pus and discharge obtained from 23 patients was sent for gram staining which showed gram positive cocci in 13 patients and gram negative rods in 1 patient. Culture revealed Staphylococcus aureus in 18, E.col in 2, klebsiella in 1 patient and was sterile in 2 patients. 

Drugs tested for Staphylococcus aureus were penicillin, methicillin, oxacillin, amoxicillin-clavulanate, cefazolin, cefoxitin, macrolides (erythromycin, azithromycin), clindamycin, clarithromycin, aminoglycosides (amikacin, gentamicin, tobramicin) Fluoro-quinolones (ciprofloxacin, ofloxacin, levofloxacin), fusidic acid, vancomycin and teicoplanin. Most strains were resistant to macrolides and penicillins. 15 were methicillin sensitive and 3 were resistant but were sensitive to amikacin, ofloxacin and vancomycin.

Gram negative rods were tested for penicillins and cephalosporins (penicillin, ampicillin, ampicillin-sulbactum, amoxicillin-clavulanate, ceftazidime, ceftriaxone, cefoperazone, cefpime), ticarcillin- clavulanate, piperacillin-tazobactum, aminoglycosides (amikacin, gentamicin, tobramicin) Fluoroquinolones (ciprofloxacin, ofloxacin, levofloxacin) carbapenems (imipenem, meropenem) and cotrimoxazole. These were sensitive to carbapenems, aminoglycosides, chloramphenicol, quinolones, piperacillin-tazobactum and cefoperazone-sulbactum, but were resistant to cephalosporins including third generation cephalosporins.

Seven of the patients had been pretreated with oral amoxicillin, cefpodoxime, 5 of these developed breast abscess. 2 patients had been managed elsewhere with needle drainage plus oral antibiotic one of these had extensive surrounding cellulitis with collection extending into the axilla and chest wall (Fig. 1). Patients responded well to open drainage via a stab incision away from the breast mound, 4 patients were managed by repeated needle aspirations. IV antibiotics were prescribed in all patients, on an average IV antibiotic was prescribed for 2-5 days and oral antibiotics were continued for 7-14 days. The baby with klebsiella received IV antibiotics for 7 days. 4 patients with systemic features or extensive local disease received 2 drugs a penicillin/cephalosporin and amino glycoside. One patient had recurrent staphylococcal infections while on follow-up. No long term follow-up is available as of now.

**Figure:**
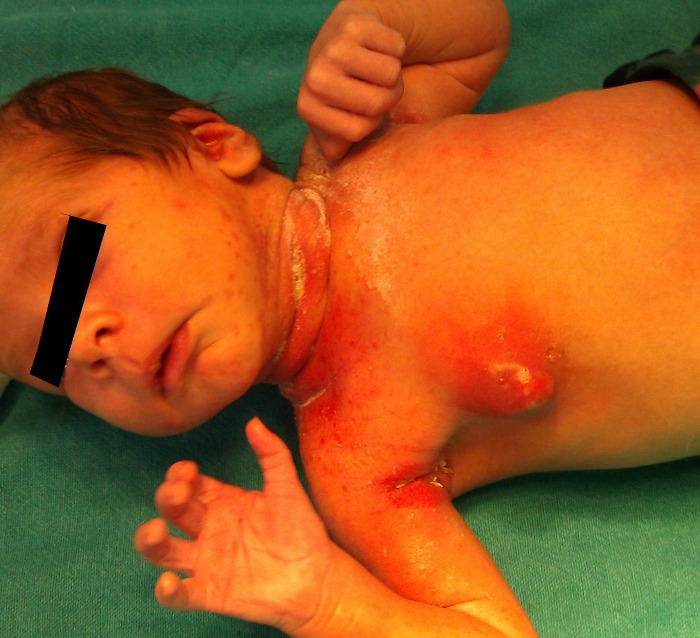
Figure 1: Neonatal breast abscess with extensive cervical and axillary pustulosis.

## DISCUSSION

That the disease was found only in term and late pre term babies is in agreement with other studies by were in no preterm babies were found to suffer from this condition. (3, 4,6) It is only in the later part of gestation that the breast is well developed and exposed to the hormonal influences. (2,3) The age range was 6-48 days only 1 child had mastitis in the first week though physiological hypertrophy is most often seen in the first week. Only reassurance and parental counseling is required. (1) Mastitis peaked in the 2nd week and no breast abscess was noted till the 3rd week with a peak in the 4th week, thus with early pickup of cases and prompt treatment abscess formation may be avoided. Ruwaili observed mastitis in the 2nd to 3rd week, breast abscess in the 3rd to 4th week, others authors have made similar observations. (3,4,6) A male: female ratio of 1:2 was observed other authors have observed ratios of, 1:5 Stricker et al, 1:1.5 Brett et al, 1:3.5 Ruwaili. . (3,4,6) Later in life there is a preponderance of female involvement with a ratio of 1:2.5 in babies with abscess peaking in the 4th week. Similar increase in the sex ratio with increasing age is observed by other authors. (2,6) 

All the babies were breast fed, however breast milk microbiological study was not done in our study though transmission of disease via breast milk has been observed by some authors.(9) Breast massage to express the secretions and fomentation to decrease the mass has been implicated by several authors in causation of mastitis. (10) Massage had been done in 15 (47%) of the affected babies in our study, reflecting the strong bias towards expressing milk from the neonatal breast especially the male babies, this probably affected the sex ratio in our study, with the ratio being 1:1.4 in the early onset mastitis group. Of the thirty two patients included in the study, 20(63%) had frank breast abscess similar ratios of 67% by Stricker et al, 52% by Brett et al, and 50% by Ruwaili were seen. (3,4,6) A relatively higher ratio was observes due to delayed referral, pretreatment with oral antibiotics, attempted needle aspiration and the practice of breast massage.

The babies were generally well with only 19% having systemic signs or symptoms Brett et al observed systemic features in 14%, Ruwaili in 8% and Stricker in 28%. (3,4,6) Though other authors have reported neonatal mastitis to be associated with suppurative lesions elsewhere in the body. (11,12) Associated skin rash pustulosis, which occurs early in life seen in 38% patients was a major predictor of severe disease including bilateral disease. Later on in the study any patient with skin pustulosis was advised strict treatment with oral and topical agents, the frequency of mastitis in the thus treated children was found to decrease.(13)

Considering that the babies were otherwise well hence besides complete, differential blood counts and CRP levels more extensive workup was not sought. Our decision was supported by clinical results and similar observations have been made by other authors. (6, 13-15) The Leucocytosis or CRP positivity did not affect the treatment plan much. (6, 13) 

Gram staining of the discharge or pus was positive in 14 out of 23 specimens (60%) Staphylococcus aureus had a higher pickup rate 13 out of 18 (72%). However the practice is not recommended by all authors.(6, 13) Usually across studies Staphylococcus aureus stands out as the most important bacterium involved in our study it was responsible for 78% of the cases, other studies have shown percentages of 100% Ruwaili, 85% Efrat et al, 69% Brett et al, 58% Stricker et al.(3,4,6) Other bacteria, gram negative rods, anaerobes have been variously reported and are consistent with our observations.(6, 15-17) The role of culture sensitivity is accepted and recommended by all authors for guiding antimicrobial therapy especially in case of a recurrence or undue prolongation of illness.(3, 5, 6, 13)

Pretreatment with oral antibiotics was seen in 7 patients 5 of which developed breast abscess similar observation was made by Stricker et al.(3) The reason for failure of oral treatment was twofold poor compliance once administered on out-patient basis and poor bioavailability of oral drugs in the neonatal period.(13,18) Needle drainage of abscess alone is recommended by Efrat et al, but in our population needle drainage had singularly poor results and lead to worsening of disease.(5) However, repeated sittings of needle drainage with IV antibiotics practiced in 20% patients had good results and none of these needed drainage. 80% patients with abscess were managed by open incision drainage with IV antibiotics with satisfactory outcome. All patients in our study were put on IV antibiotics for a period of 2-5 days, similar practice is recommended by Efrat et al, though, other authors have used IV antibiotics in lesser number of patients Brett et al in 38%, Ruwaili in 80%, Stricker in 78%, the duration of IV use has been similar. (3-6) Oral antibiotics were continued for 7-14 days with amoxicillin-clavulanate, cephalexin, linezolid and clindamycin being the drugs used. Quinolones were used for gram negative rods after completing IV therapy. Shift to oral therapy was based on clinical improvement as in other studies.(3-6) There was no recurrence of illness.

## Recommendation

From our study we can conclude neonatal breast hypertrophy is a benign condition and settles spontaneously in most babies. The practice of breast massage should be condemned and parents should be appropriately counseled. Any skin rash especially pustulosis should be taken seriously and appropriate therapy oral or topical instituted. Intravenous antibiotics should be used for this condition guided by gram stain or culture sensitivity once available. Empirically a drug with good anti-staph cover may be instituted till appropriate reports are available. Therapy can be shifted to oral drugs once clinical improvement is seen. Incision drainage gives uniformly good results, though; multiple sittings of needle drainage may obviate the need for incision drainage.

## Footnotes

**Source of Support:** Nil

**Conflict of Interest:** None

